# The in vitro toxicity evaluation of halloysite nanotubes (HNTs) in human lung cells

**DOI:** 10.1007/s43188-020-00062-1

**Published:** 2020-10-13

**Authors:** Dorota Sawicka, Lidia Zapor, Luiza Chojnacka-Puchta, Katarzyna Miranowicz-Dzierzawska

**Affiliations:** Central Institute for Labour Protection, National Research Institute, Czerniakowska 16, 00-701 Warsaw, Poland

**Keywords:** Cytotoxicity, Halloysite nanotubes, Holo-tomographic microscopy, Lung cells

## Abstract

Halloysite nanotubes (HNTs) have been increasingly used in many industrial and biomedical fields. Therefore, the assessment of risk and consequences of exposure to HNTs is very important to better protect human safety. This study aims to investigate the short- (24 or 72 h) and long-term (7 days) cytotoxic effects of HNTs at doses 10–200 µg/mL on human alveolar carcinoma epithelial cells (A549) and human bronchial epithelial cells (BEAS-2B). The effect of HNTs on cell viability, apoptosis, cell proliferation, oxidative/antioxidative status and cell morphology was evaluated. Our results showed that cytotoxicity of HNTs is dependent on dose, cell model and time of exposure. During the time of exposition toxic effects were intensified. To the best of our knowledge, this is the first study to use holo-tomographic microscopy (HTM) to visualise changes in cell morphology due to exposure from HNTs. We observed cells contraction, changes in the size and shape, cell surface folding and cytoplasmic vacuolization, peripheral arrangement of cell nuclei and even increase number of nucleus, which undoubtedly confirmed cytotoxic effect of HNTs at low doses (5 µg/mL and 25 µg/mL). Our results demonstrated that HTM technique provides a new insight into the assessment of HNTs toxicity. Further studies with different cell models are recommended to assess the toxic effect of HNTs on whole human body.

**Electronic supplementary material:**

The online version of this article (10.1007/s43188-020-00062-1) contains supplementary material, which is available to authorized users.

## Introduction

Halloysite (Al_2_Si_2_O_5_(OH)_4_·*n*H_2_O) is two-layered aluminosilicate with a unique hollow tubular structure and high aspect ratio. The size of halloysite nanotubes (HNTs) varies within 1–15 µm and 10–150 nm of inner diameter. HNTs occurs naturally, and it is chemically similar to another clay mineral – kaolin [1, 2]. The characteristics feature, such as unique tubular structure, high aspect ratio, nanoscale lumens, cheap and abundant availability make this nanomaterial useful in many applications [3]. HNTs have huge potential in biomedicine, including drug and gene delivery vesicles, tissue engineering, bone implants, ultrasound contrast agents, teeth fillers, cancer and stem cells isolation and cosmetics [4–8]. Furthermore, it is usually used in the fabrication of high-quality ceramic white-ware and for preparing polymer-based composites (as nanofiller), remediation of environmental contaminants, storage of molecular hydrogen, catalytic conversion and processing of hydrocarbons, as nano additives for pigments and many others [9–12].

Similarity of HNTs tubular structure to carbon nanotubes (CNTs) may potentially cause the same toxicity such for CNTs, which include pulmonary inflammation and acute phase response following pulmonary exposure and even tumour changes in the human respiratory system [13–16]. Taking into consideration many applications of HNTs, the evaluation of HNTs toxicity is crucial for workers safety. The information on toxicological effects of HNTs on human health is limited [1], therefore, there is an emerging need to investigate the potential risk and consequences of effects of exposure to HNTs.

This study evaluates the effect of HNTs at doses 10–200 µg/mL on cell viability and apoptosis using human alveolar carcinoma epithelial cells (A549) and human bronchial epithelium cells (BEAS-2B) after short- (24 or 72 h) or long-term (7 days) exposure to HNTs. The ability of cells to proliferate and oxidative/antioxidative status were also examined. We also observed the morphological changes in cells exposed to low doses of HNTs (5 µg/mL and 25 µg/mL) using the holo-tomographic microscopy (HTM).

## Materials and methods

### Preparation and characterisation of HNTs

The morphology of HNTs (Sigma-Aldrich, St. Louis, MO, USA) was characterised using scanning electron microscopy (SEM, Zeiss Ultra Plus, Germany). The particle size and size distribution of HNTs were analysed using the nanoparticle tracking analysis (NTA) (NS500, Malvern Instruments, Amesbury, UK). The HNTs were suspended in culture media used for culturing BEAS-2B cells or for A549 cells. The suspensions were sonicated in an ice bath at high energy level of 420 J/cm^3^ (60 s with amplitude 90%) (Sonica Q 700, Qsonica LLC, USA) and stirred for 1 min to ensure homogeneity before measurements at three time points: after 5 min, 24 h and 48 h. Such prepared suspensions were loaded into the laser module sample chamber and viewed in close proximity to the optical element. Particles in the liquid sample which passed through the beam path were seen as small points of light moving rapidly under Brownian motion, allowed information on particle properties. Particles moves were registered by CCD camera, due to scattering of laser light beam. Recorded video capture enabled latter particles identification and movement route analysis. Thanks to simultaneous video-tracking of single particles we obtained high-resolution data about particle size distribution, such as mean size and mode size. The "mean value” was the arithmetic mean of particle size distribution and the "mode value” represents the particle size most commonly found in the particle size distribution. The data was captured and analysed using the software NTA 2.3 (Malvern). The same procedure of preparation HNTs was applied to all cytotoxicity assays.

### Cell culture and treatment

The A549 cells (CCL185) and BEAS-2B cells (CRL-9609) were obtained from American Type Culture Collection (ATCC, LGC Standards, UK). The A549 cells were cultured as a monolayer in an F12K medium (Gibco, Invitrogen, Carlsbad, CA, USA) supplemented with 10% foetal bovine serum (FBS, Gibco) and 1% antibiotic-antimycotic (Sigma-Aldrich) in sterile tissue culture flasks (Nunc, Roskilde, Denmark). BEAS-2B cells were cultured in the LHC-9 serum-free medium (Thermo Fisher Scientific MA, USA) containing 1% antibiotic-antimycotic (Sigma-Aldrich). Both cell lines were grown at 37 °C and in a humidified atmosphere (5% CO_2_). The cells were passaged before reaching 90% confluence. To detach cells from the culture plates, 0.25% trypsin-EDTA (Gibco) was used. Additionally, cells were screened for *Mycoplasma* sp. infection using MycoAlert™ PLUS Mycoplasma Detection Kit (Lonza, Walkersville, Inc.).

### Cytotoxicity assays of HNTs

#### MTT assay

The in vitro cytotoxicity of HNTs was evaluated using MTT assay as described by Mossman [17]. This assay is based on measuring mitochondrial dehydrogenase activity living cells. In brief, BEAS-2B cells or A549 cells were seeded at a density of 10,000 cells/well in a 96-well culture plate (Nunc) and cultured overnight (37 °C, 5% CO_2_). After this period, non-attached cells were aspirated followed by the addition of HNTs suspension at different concentrations (10–200 µg/mL). The plates were then incubated for 24 or 72 h. Then, the medium with HNTs was removed and cells were washed with phosphate buffer saline (PBS) (Gibco). Subsequently, the medium containing MTT (3-(4.5 dimethylthiazol-2-yl)-2.5 diphenyltetrazolium bromide, 5 mg/cm^3^, Sigma-Aldrich) was added and the plates were incubated for 3 h and observed until a purple coloured formazan product was formed. This product was dissolved in dimethyl sulphoxide (Gibco) and plates were shaken. Then the absorbance was measured at 570/620 nm using SYNERGY 2 (BioTek Instruments, Inc.) microplates reader. Cytotoxicity tests were performed in at least three independent replications. Based on the absorbance values obtained in MTT test, the viability ratio of cells exposed to the different concentration of HNTs, i.e. the percentage of viable cells compared to control was calculated, followed by the calculation of concentrations of HNTs that reduced cell viability by 50% compared to control (IC_50_).

#### Clonogenic assay

Clonogenic assay also known as colony-forming efficiency assay (CFEA) is based on the ability of a single cell to grow into a colony [18]. A colony is defined to consist of at least 50 clones of one cell (which corresponds to six mitotic divisions). This test detects the number of cells that retained the capacity for producing a large number of progenies after HNTs treatment [19].

The clonogenic assay was conducted according to the procedure described by Franken et al. [20] and adapted from Kruszewski et al. [21]. Briefly, cells in the exponential growth phase (BEAS-2B cells or A549 cells) were harvested and seeded in Petri dish 60 × 15 mm (21 cm^2^) (Iwaki Cell Biology, Japan) at a density 500 cells/dish in 5 mL of medium containing HNTs in appropriate concentrations. Each experiment was performed at least in three independent replicates. Cells were exposed to HNTs (25–200 µg/mL) for 7 days. After this period, the medium was removed, and cells were washed with PBS. Then, colonies were fixed with ethanol (Sigma-Aldrich), stained with Giemza solution (0.4%, Sigma- Aldrich) and counted using a stereomicroscope (IUL, Spain). The plating efficiency (PE) and surviving fraction (SF) was calculated, as below:

$${\text{PE}} = ({\text{number of colonies formed/number of cells seeded}}).$$

$$\begin{aligned}& {\text{SF}} = ({\text{number of colonies formed after treatment/}}\\& \quad{\text{number of cells seeded}} \times {\text{PE}}). \end{aligned}$$

The PE ratio for A549 and BEAS-2B cells calculated from three independent experiments was above 0.6.

#### TOS/TAS assay

The total oxidative status (TOS) of cells was determined using the PerOx (TOS/TOC) kit (Immundiagnostic AG, Germany), that measures a total level of lipid peroxides in cells. The total antioxidative status (TAS) of cells was determined in the reaction of antioxidants with a pre-defined amount of exogenous hydrogen peroxide (H_2_O_2_) using the ImAnOx (TAS/TAC) kit (Immundiagnostic AG). Both tests were performed according to the manufacturer’s protocols. In brief, BEAS-2B cells or A549 cells were seeded in 96-well microplates and exposed to HNTs (100 and 200 µg/mL) for 24 h. Then, the cells were washed twice with 100 µl of PBS (Gibco) and lysed by freezing at − 80 °C (in the three thaw/freeze cycles). Before the evaluation of the oxidative/antioxidative status, cell lysates were sonicated for 30 min and then centrifuged (10.000×*g* for 5 min). The absorbance was measured at the wavelength of 450 nm using SYNERGY 2 microplates reader (BioTek Instruments). Each experiment was performed in at least three independent replications. The obtained values were converted to 1 mg of protein in the sample.

#### Caspase 3/7 assay

The caspase 3/7 activity was evaluated using an automated in-incubator fluorescence microscope IncuCyte S3 (Live-Cell Analysis System, Sartorius, Michigan, MI, USA). After labelling cells with reagent, this system allows the determination of active caspase 3/7, which is expressed in apoptotic cells. The inert, reagent freely crosses the cell membrane, and after cleavage by caspase 3, releases a green DNA-binding dye. The green fluorescence of the cleaved substrate can be measured at an excitation maximum of 500 nm and an emission maximum of 530 nm.

BEAS-2B cells or A549 cells were seeded at a density of 10,000 cells per well in 96-well plates (Nunc) and incubated overnight at 37 °C in a humidified atmosphere (5% CO_2_). Next, cells were exposed to different concentrations of HNTs (10–200 µg/mL) for 24 or 48 h. Subsequently, we replaced medium with fresh medium containing 1x IncuCyte Caspase- 3/7 Apoptosis Assay Reagent (Sartorius). Before start scanning, plates were incubated (37 °C, 5% CO_2_) for 15 min. As a positive control we used cells treated with staurosporine (37 nM, Sigma-Aldrich). At each time point, five images were taken per well in both brightfield and FITC channels. Images were analysed for the number of green objects (fluorescing cells) per well by the algorithm in the IncuCyte S3 Software (v2018B). Each experiment was performed in at least three independent replications.

### Visualisation of morphological changes in exposed cells via HTM

For the preparation of samples, BEAS-2B cells or A549 cells were seeded into 35-mm culture dishes (IBIDI, Gräfelfing, Germany) at a density 20,000 cells/dish and incubated for 24 h. Then, cells were treated with HNTs at doses 5 and 25 µg/mL and incubated for 24 or 72 h. The dishes containing untreated cells (control) or exposed cells were placed on the tomographic microscope (3D Cell Explorer, Nanolive S.A., Lausanne, Switzerland) and three-dimensional tomographic images (z-stacks) were created. Further, post-processing steps such as background reduction and contrast enhancement were applied to the final figures using STEVE software (Nanolive).

### Statistical analysis

Statistical analysis was carried out using the Statistica, version 7.1. The IC_50_ values obtained based on the analysis of a series of dose–response curves were evaluated using non-linear regression analysis at 95% confidence interval (concentrations reducing cell viability by 50% as compared with the control). CFEA, NTA results, activity of caspase 3/7 and TOS/TAS data were analysed using Student’s *t*-test. A value of *p* < 0.05 indicates a statistically significant difference. The data were presented as the mean ± standard deviation (SD).

## Results

### Characterisation of HNTs

**Fig. 1:**
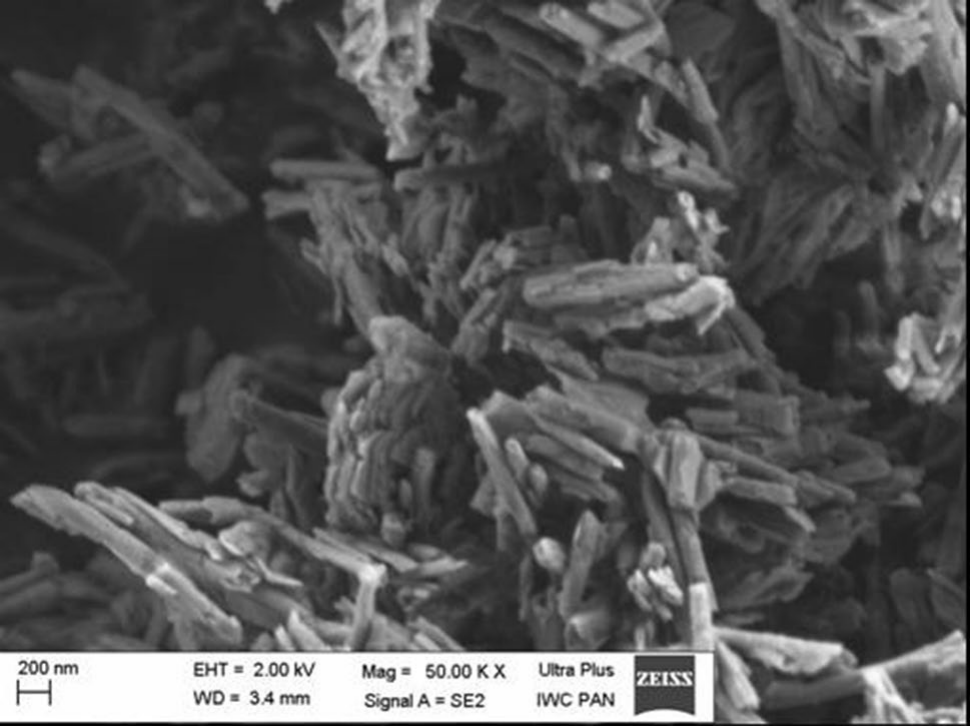
The characterisation of HNTs. The morphology of HNTs was characterised using scanning electron microscopy (SEM). Magnification and scale are presented on the image

**Fig. 2:**
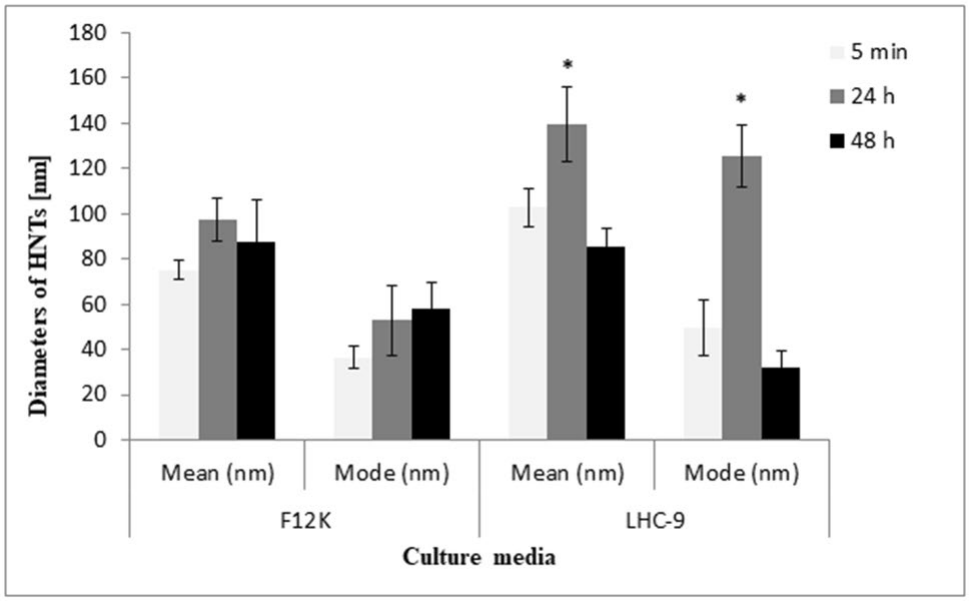
Analysis of HNTs size distribution. The diameters of the HNTs were measured in the F12K containing 10% FBS or LHC-9 media after 5 min, 24 and 48 h using. The graph represents high-resolution data about particle size distribution, such as mean size and mode size. Data represent the mean and mode value ± SD. *Significant differences at *p* < 0.05

The scanning electron microscopy (SEM) images were carried out to characterise the morphology and detailed structure of HNTs. As illustrated in Fig. 1, the SEM image analysis confirmed tubular morphology of HNTs with length > 1000 nm and diameter circa 100 nm. The NTA analysis showed that after 24 h, diameters of nanotubes were different in LHC-9 medium and F12K medium containing 10% FBS. After 48 h, we observed decrease in diameters of nanotubes in both media (Fig. 2). The mode values obtained after 24 h, indicated a large share of nanotubes below 100 nm in the F12K medium supplemented with 10% FBS compared with LHC-9 medium.

### Cell cytotoxicity assays

#### MTT assay

**Fig. 3:**
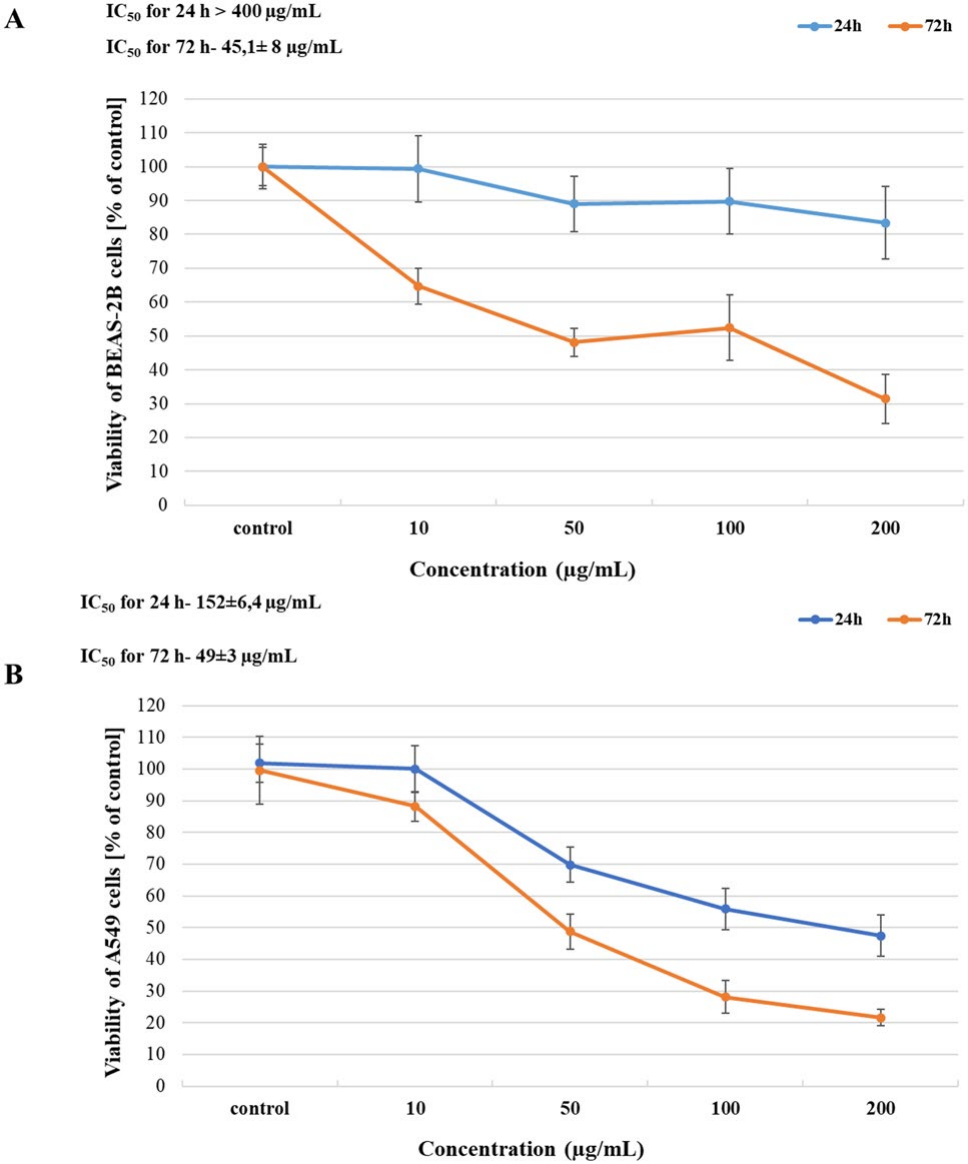
Analysis of HNTs cytotoxicity. The BEAS-2B cells (**a**) and A 549 cells (**b**) were examined by MTT assay after 24 and 72 h of exposure to HNTs (10–200 µg/mL). Data are presented as the mean ± SD

We analysed the cytotoxicity profile HNTs on A549 and BEAS-2B cells using MTT assay and evaluated cytotoxicity based on the IC_50_ values. The results showed that HNTs cytotoxic potency was dependent on dose, cell model and time of exposure. After 24 h of exposure, the IC_50_ of HNTs in A549 and BEAS-2B cells was 152 ± 6.4 µg/mL and > 400 µg/mL (extrapolated value), respectively. However, in IC_50_ values decreased to 49 ± 3 µg/mL and 45.1 ± 8 µg/mL, respectively, in A549 and BEAS-2B cells after 72 h of exposure (Fig. 3a, b).

#### Clonogenic assay (CFEA)

**Fig. 4:**
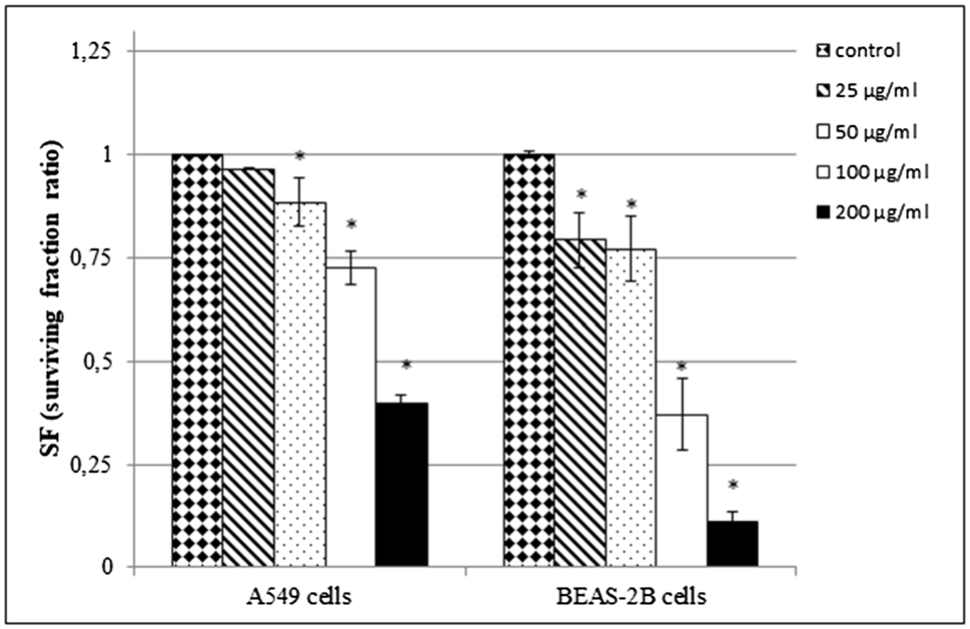
The ability of A549 or BEAS-2B cells to form colonies after exposure to HNTs. The cells were treated with different concentrations of HNTs (25–200 µg/mL) for 7 days. Data represent the mean ± SD. *Significantly different from untreated control (*p*< 0.05)

As shown in Fig. 4, HNTs to significantly affected the clonogenic survival and cell proliferation in a dose-dependent manner after 7 days of exposure. After exposing A549 cells to HNTs at dose 50–200 µg/mL, we observed a significant difference in SF value compared with control. The ability to form colony significantly decreased in BEAS-2B cells after exposure to HNTs at doses 25–200 µg/mL (Fig. 4).

#### Assessment of total oxidative/antioxidative status (TOS/TAS) of cells exposed to HNTs

**Fig. 5:**
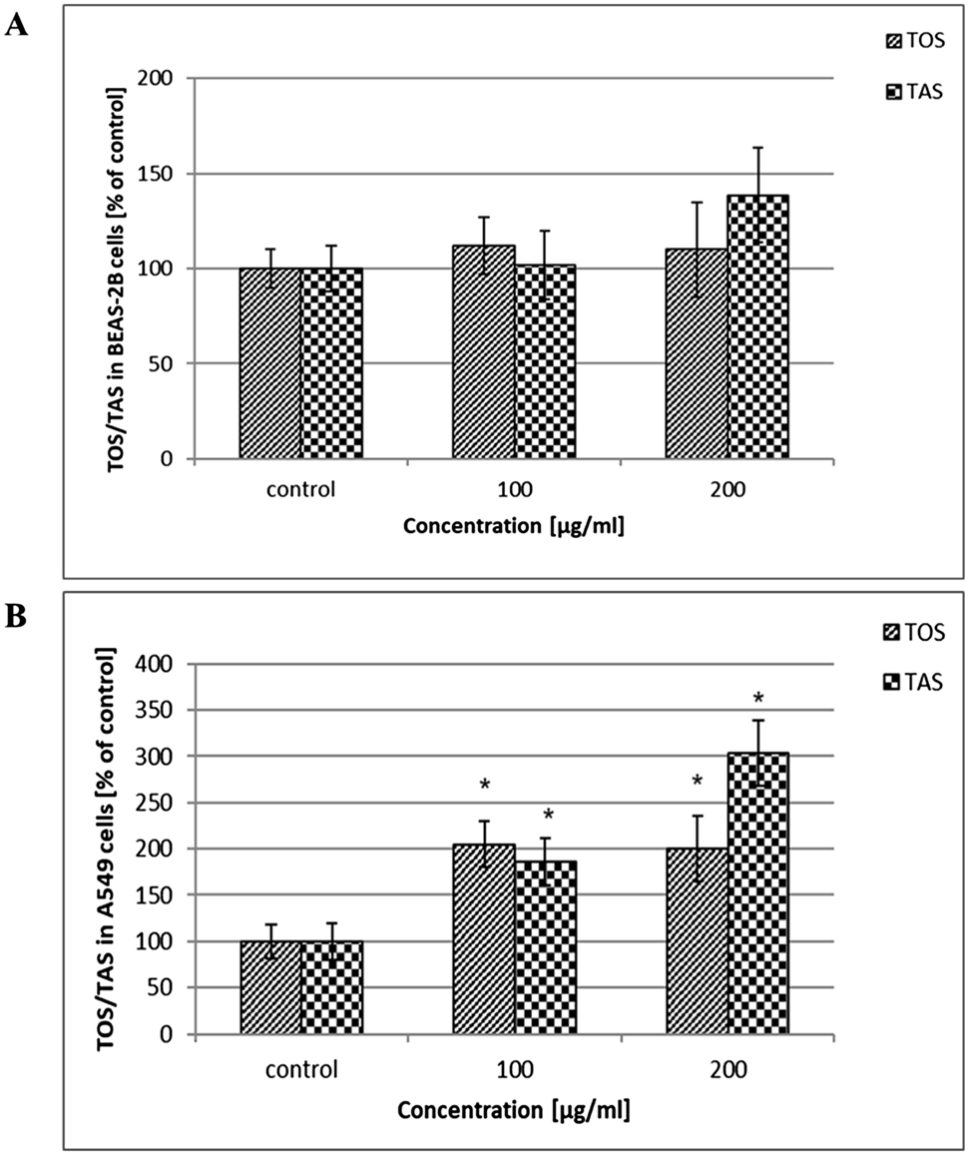
Analysis of total oxidative status (TOS) and total antioxidative status (TAS) in cells treated with HNTs. The BEAS-2B cells (**a**) and A549 cells (**b**) were exposed to HNTs (100–200 µg/mL) for 24 h. Data represent the mean ± SD. *Significantly different from untreated control (*p* < 0.05)

The total oxidative and antioxidative status (TOS/TAS) of the cells exposed to HNTs (100–200 µg/mL) after 24 h is presented in Fig. 5a, b. In exposed BEAS-2B cells, we did not observe significant changes in the TOS/TAS level compared with the control (Fig. 5a). However, we noticed a slight increase in TAS level after exposure cells to 200 µg/mL of HNTs. The change in TOS/TAS level in A549 cells was dose dependent. We observed an increase in TOS in A549 cells exposed to HNTs at concentration 100 µg/mL, but it was accompanied by an increase in TAS. The TAS level after exposure A549 cells to 200 µg/mL of HNTs was higher than TOS (Fig. 5b).

**Fig. 6:**
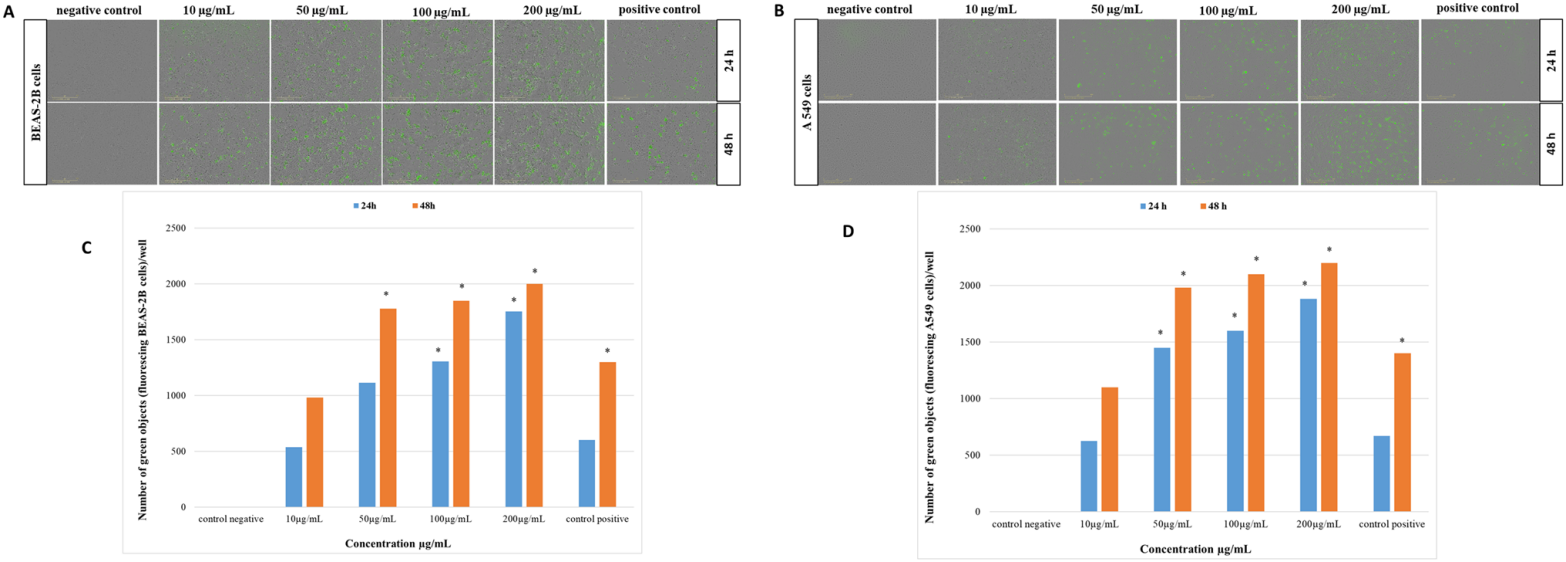
Analysis of Caspase 3/7 activity in cells exposed to HNTs. BEAS-2B cells (**a**) or A549 cells (**b**) were treated with HNTs (10–200 µg/mL) and staurosporine (37 nM) for 24 or 48 h. Fluorescence images of over time were obtained with the use of image-based live-cell analysis system (IncuCyte S3). A caspase 3/7 signal (green) represents apoptotic cells. The representative pictures showed cells with a tenfold lens. **c**, **d** Graphic presentation of the caspase 3/7 activity analysis results in BEAS-2B cells and A549 cells, respectively. ± SD not visible. *Significantly different from negative control (*p* < 0.05)

#### Caspase 3/7 assay

We determined the activity of caspase 3/7 in BEAS-2B cells or A549 cells using IncuCyte S3 after 24 or 48 h of treatment with HNTs (10–200 µg/mL) and 37 nM of staurosporine (positive control). In both cells, we observed an increase in the number of apoptotic cells in a dose- and time-dependent manner. Compared with negative control (untreated cells), the number apoptotic A549 cells significantly increased after 24 or 48 h of treatment with HNTs (50 µg/mL) (Fig. 6b, d). The caspase 3/7 activity in BEAS-2B cells significantly dependent on 24 h of exposure on 100 µg/mL of HNTs. We observed increase number of apoptotic BEAS-2B cells after 48 h of exposure on HNTs in doses from 50 µg/mL (Fig. 6a, c).

### Visualisation of morphological changes in exposed cells via HTM

**Fig. 7:**
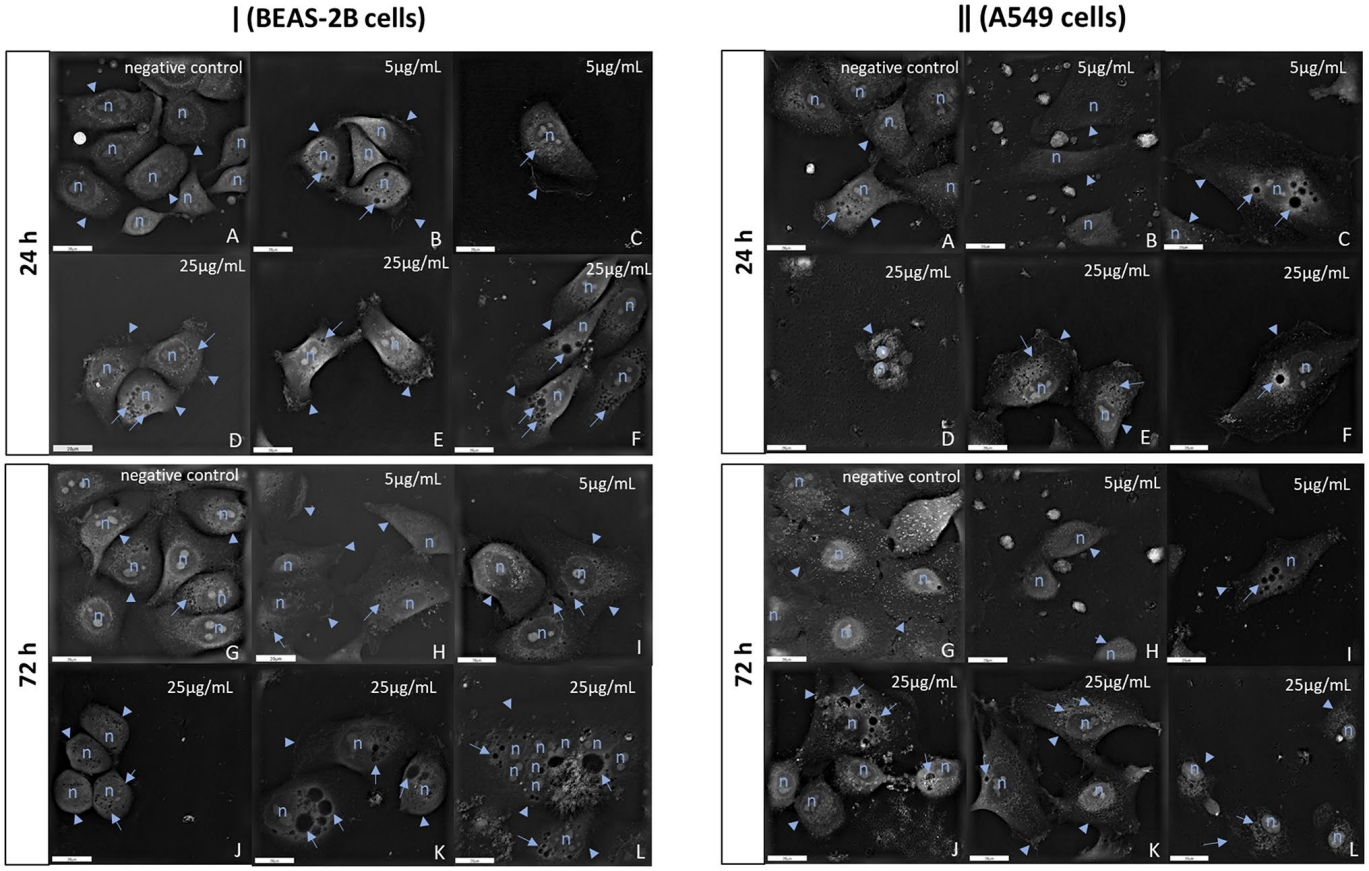
Dose and time dependent morphological changes in cells after treatment with HNTs. Representative HTM images of BEAS-2B cells [ǀ]) and A549 cells [ǁ] incubated for 24 or 72 h with 5 or 25 µg/mL of HNTs. **a**, **g** Represent negative control (untreated cells) after 24 and 72 h, respectively. **b**–**f** Represent cells after 24 h of treatment with HNTs 5 µg/mL and 25 µg/mL, respectively. **h**–**l **Represent cells after 72 h of exposure to HNTs 5 µg/mL and 25 µg/mL, respectively. HTM images of cells showed (n) nuclei, numerous of cytoplasmic vacuoles (arrows) and cytoplasmic membrane (arrowheads). Description of changes is presented in the text. Scale bar 20 µm

Treatment of BEAS-2B or A549 cells with HNTs at low doses 5 and 25 µg/mL, caused morphological changes after 24 or 72 h. The untreated cells (controls) had no alteration in cell morphology or any membrane damages (Fig. 7a, g). After 24 or 72 h of exposure, we observed cell contraction and changes in the size and shape in both BEAS-2B or A549 cells (Fig. 7 [ǀ] b, d–e, i–k and Fig. 7 [ǁ] b, d, h, j, l). Also, we observed the loss of cell-cell contact in both treated cells compared with controls (Fig. 7). The result of the exposure of BEAS-2B or A549 cells to both doses of HNTs was also the characteristic cell surface folding (Fig. 7 [ǀ] b–f, h and Fig. 7 [ǁ] e–f, k) and cytoplasmic vacuolisation (Fig. 7 [ǀ] b–f, h–l and Fig. 7 [ǁ] c, e–f, i–l). During the treatment, the degree of cytoplasmic vacuolisation was increased. After 72 h of exposure BEAS-2B cells to 5 and 25 µg/mL of HNTs, we observed peripheral arrangement of cell nuclei (Fig. 7 [ǀ] h) and even increased number of nuclei (Fig. 7 [ǁ] l), respectively.

## Discussion

In recent years, the application nanomaterials has increased in many fields including industrial, medicinal and everyday objects. Although nanomaterials have potential benefits, their interaction with biological systems may cause unpredictable risk to human life. Halloysite is a natural nanosized tubular clay mineral that has many potentially important applications, such as inter alia environmental sciences, biomedicine, waste-water treatment, nanoelectronics, cosmetics, dye removal, fabrication of nanocomposites and forensic sciences [2]. Therefore, assessment of toxicity of HNTs is necessary to assess the human risk, especially in the working environment from a safety point of view. Our study focused on two important aspects. Firstly, we evaluated the cytotoxic effects in A549 cells and BEAS-2B cells after short- (24 or 72 h) and long-term (7 days) treatment with HNTs. Secondly, we visualized the morphological changes in cells exposed to low doses of HNTs using HTM.

The cytotoxicity of nanoparticles (NPs) depends on their properties such as shape and particle size, agglomeration, surface charge and modification, mechanism of cellular uptake and toxicity response [22]. Characterisation of nanomaterials is the first step in the study of in vitro toxicity, especially evaluation of the “real-time” particles size and size distribution in the media used for cell culture. We characterized HNTs for size and particle size distribution using the NTA technique and primary size and morphology using SEM [23]. Our findings are in accordance with those of Gaaz et al. [24], we confirmed tubular morphology of HNTs with particles length and diameter circa above 1000 nm and 100 nm, respectively (Fig. 1). The mode values evaluated after 24 h indicated that large share the particles in the F12K medium containing 10% FBS were below 100 nm, which might have influenced the IC_50_ value. We also observed that after 24 h, diameters of the particles were different in medium LHC-9 and F12K containing 10% FBS and found to decrease over time (Fig. 2), which was in correlation with higher cytotoxicity observed after long time exposure (Fig. 3).

The toxicity tests used for screening of the NPs are mainly based on measuring the activity of living cells via mitochondrial dehydrogenase activity (MTT) [25], cell membrane integrity (NRU, trypan blue, propidium iodide) [26], apoptosis [27, 28], cytokine [29], oxidative stress [30, 31], ability to proliferate [32, 33] and DNA damages [34, 35]. Vergaro et al. [36] observed that the growth inhibition in MCF-7 and Hela cells exposed to HNTs was concentration dependent. They demonstrated that cell viability was 70% at doses of HNT up to 75 µg/mL. The viability of both cells was decreased with the increase of HNTs concentration up to 1000 µg/mL.

We also evaluated the cytotoxicity of HNTs using MTT assays. The IC_50_ value of HNTs in A549 cells and BEAS-2B cells after 24 h of exposure was 152 ± 6.4 µg/mL and > 400 µg/mL, respectively. As shown in Fig. 2, the diameter of HNTs decreases over time in media for both cells, which may influence the cytotoxic effect of HNTs. After 72 h, we observed a decrease in IC_50_ values in A549 and BEAS-2B cells to 49 ± 3 µg/mL and 45.1 ± 8 µg/mL, respectively (Fig. 3a, b). Similarly, Verma et al. [37] also demonstrated that HNTs did not cause significant cytotoxic effect after 24 h of exposure in A549 cells at concentration up to 100 µg/mL. These results were obtained using cell-based automated high content screening in combination with real-time impedance sensing. Lai et al. [38] have evaluated HNTs toxicity in co-culture of intestinal cells (Caco-2/HT29-MTX) using LDH leakage, XTT assay and permeability assay. They did not observe toxic effect after 6 h of exposure to HNTs at doses 0-100 µg/mL. Thus, the sensitivity to HNTs is dependent on dose, cell model and time of exposure.

Ahmed et al. [39] used one of the cell proliferation assays (WST-1) to examine the toxic potential of HNTs and showed that viability of hepatocellular carcinoma (HepG2) cells and colorectal carcinoma (HCT116) cells decreased in a dose-dependent manner with cytostatic activity at 250–500 µg/mL and cytotoxicity at 1000 µg/mL. Another method to examine cells ability to proliferate is clonogenic assay (CFEA). The advantage of this test is that it does not require any cellular dyes, which may interact with tested NPs and lead to invalid results [19, 40–42]. The CFEA assay allows to identify compounds which give rise to “mitotic death” as a result of DNA damage or apoptosis [19] and to assess the possible consequence of long-term exposure (7–10 days) at sub-lethal doses of NPs. We used CFEA to evaluate ability of A549 and BEAS-2B cells to form colony after 7 days of exposure to HNTs at doses 25–200 µg/mL. A significant difference in SF value was observed in A549 after using HNTs in the dose range 100–200 µg/mL. The cell proliferation was significantly decreased after exposure of BEAS-2B cells to HNTs at doses 25–200 µg/mL (Fig. 4), which confirmed that toxicity of HNTs increased overtime of exposition.

It has been reported that NPs caused mitochondrial dysfunction by increasing levels of mitochondrial reactive oxygen species (ROS) [43, 44]. Because ROS are responsible for oxidative stress in cells, the determination of the total oxidative (TOS)/ total antioxidative (TAS) status is very important in toxicology research. Overproduction of ROS leads to change in cell motility, cytotoxicity, apoptosis, unregulated cell signalling, DNA damage and cancer initiation [45]. In our study, we evaluated TOS/TAS status after 24 h of exposure BEAS-2B cells or A549 cells to HNTs at doses 100–200 µg/mL. In treated BEAS-2B cells, we did not observe significant changes in the TOS/TAS level compared with control. However, we noticed a slight increase of TAS level after exposure cells to 200 µg/mL of HNTs (Fig. 5a). The TOS/TAS level in treated A549 cells was significantly dependent on the dose. After using HNTs at concentration 100 µg/mL, we observed an increase in TOS, but it was accompanied by an increase in TAS. We observed noticeable differences in the TOS and TAS levels in A549 cells after treatment with 200 µg/mL of HNTs (Fig. 5b). It was worth to notice, that level of TAS was higher than that of TOS, which may indicate that cells have defence against oxidative stress mechanism [46], which is based on the system of antioxidants enzymes and non-enzymatic antioxidant substances capable of neutralising free radicals and preventing excess production of ROS.

Another cytotoxic effect of NPs is apoptosis resulting from activation of caspase [47]. Liu et al. [48] have analysed apoptosis in A549 cells exposed to HNTs at doses 10–200 µg/mL using TUNEL test. They showed a significant effect of HNTs on the number of apoptotic cells after 12 h of exposure at a concentration from 50 µg/mL. In our study, we examined apoptosis in BEAS-2B cells or A549 cells using caspase 3/7 assay after 24 or 48 h of exposure cells to HNTs (10–200 µg/mL). Despite HNTs did not significantly decrease cell viabilities after 24 h of exposure (Fig. 3), we observed an increase number of apoptotic cells in a dose- and time-dependent manner. Compare with negative control (untreated cells), HNTs (50 µg/mL) significantly affected the number of apoptotic A549 cells after 24 or 48 h (Fig. 6b, d). The caspase 3/7 activity in BEAS-2B cells significantly dependent on 24 h of exposure of 100 µg/mL of HNTs (Fig. 6a, c). Increase number of apoptotic BEAS-2B cells after 48 h of exposure on HNTs in doses from 50 µg/mL was observed. Our result from caspase 3/7 assay and TOS/TAS assay showed that HNTs induced apoptosis independent of ROS.

Treatment of cells with NPs also caused morphological changes such as the formation of blebbed nuclei, numerous lipid droplets in the cytoplasm and increased numbers of mitochondria and cytoplasmic vacuoles containing nanoparticles [49–51]. To date, the changes in morphology of cells exposed to NPs were documented using optical microscopy, scanning electron microscopy (SEM) and transmission electron microscopy (TEM) [51–55]. SEM and TEM allow to obtain more detailed images, but require proper preparation of samples including trypsinisation, fixation or staining of cells which may affect cell morphology. To the best of our knowledge, this is the first study to use HTM for visualising cytotoxic effects of HNTs at low doses on cell morphology. It is worth to notice that this technique does not require any preparation of the sample.

We observed cell contraction in A549 and BEAS-2B cells exposed to HNTs at doses 5 and 25 µg/mL after 24 or 72 h. It might be due to the loss of intracellular waters and electrolytes and thus resulted in changes in the size and shape of the cells (Fig. 7 [ǀ] b, d–e, i–k and Fig. 7 [ǁ] b, d, h, j, l) in compare to control which had regular shape without any membrane damages (Fig. 7a, g).

During cells incubation, the loss of cell-cell contact was observed in both treated cells compared with control (Figs. 7). The characteristic of cell surface folding (Fig. 7 [ǀ] b–f, h and Fig. 7 [ǁ] e, f, k) and cytoplasmic vacuolisation (Fig. 7 [ǀ] b–f, h–l and Fig. 7 [ǁ] c, e–f, i–l) was also demonstrated after treated cells with both doses of HNTs. After 72 h of treatment BEAS-2B cells with 5 µg/mL, we observed peripheral arrangement of cell nuclei (Fig. 7 [ǀ] h). Furthermore, after 72 h of exposure BEAS-2B cells to 25 µg/mL of HNTs, we observed an increase in the number of nuclei (Fig. 7 [ǀ] l), which has been used by pathologists as a prognostic indicator of cancer [56]. Our results showed that doses of HNTs below IC_50_ caused morphological changes, that testify to adverse processes in the cells.

In conclusion, expanding application HNTs in many fields of industry or biomedicine and the associated increasing of unintentional exposure of humans to HNTs indicate that there is a need to estimate of its safety. Thus, a better understanding of HNTs cytotoxicity will allow to implement better protective measures for industrial workers. We presented that cytotoxicity of HNTs was dependent on dose, cell model and time of exposure. Furthermore, increase time of treatment with HNTs intensified the cytotoxic effects, which suggesting its potential chronic toxicity. HTM technology revealed that HNTs induce morphological changes in cells, which confirms the cytotoxic effect of HNTs even at low doses. Further studies with different cell models are recommended to assess the toxic effect of HNTs on the whole human body.

## Electronic supplementary material

Below is the link to the electronic supplementary material.

Supplementary material 1 (PNG 139.2 kb)

Supplementary material 2 (PNG 7.3 kb)

Supplementary material 3 (PNG 24.0 kb)

Supplementary material 4 (PNG 8.0 kb)

Supplementary material 5 (PNG 63.3 kb)

Supplementary material 6 (PNG 315.2 kb)

Supplementary material 7 (PNG 272.8 kb)
