# Supplementary figures and images for: The in vitro toxicity evaluation of halloysite nanotubes (HNTs) in human lung cells
Source: Toxicol Res. 2020 Oct 13;37(3):301–10. doi: 10.1007/s43188-020-00062-1 (PMC8249553; doi:10.1007/s43188-020-00062-1)

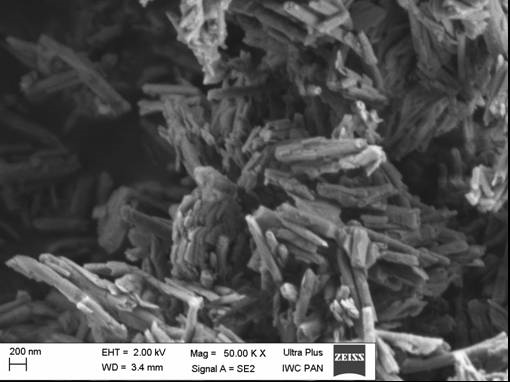

Supplement: Supplementary file 1 — Supplementary material 1 (PNG 139.2 kb) [file 43188_2020_62_MOESM1_ESM.png]

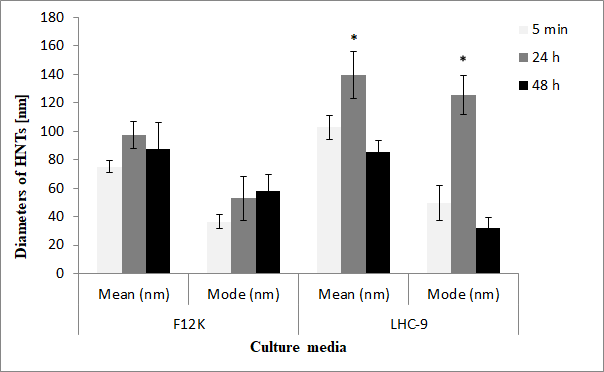

Supplement: Supplementary file 2 — Supplementary material 2 (PNG 7.3 kb) [file 43188_2020_62_MOESM2_ESM.png]

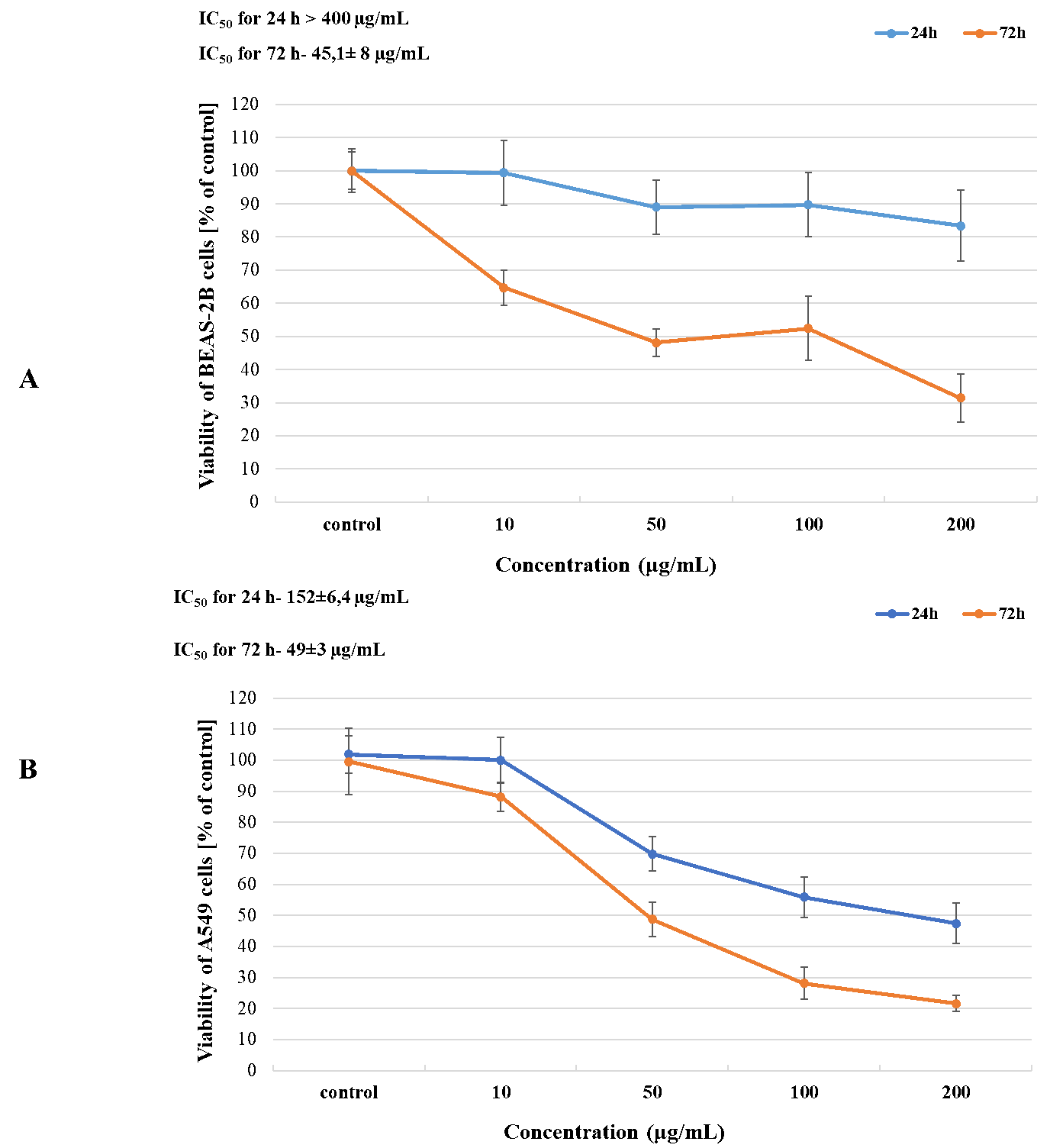

Supplement: Supplementary file 3 — Supplementary material 3 (PNG 24.0 kb) [file 43188_2020_62_MOESM3_ESM.png]

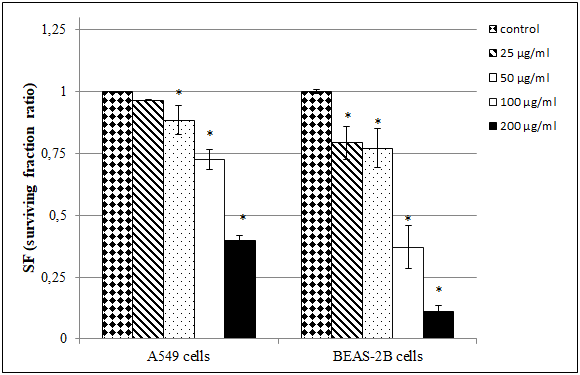

Supplement: Supplementary file 4 — Supplementary material 4 (PNG 8.0 kb) [file 43188_2020_62_MOESM4_ESM.png]

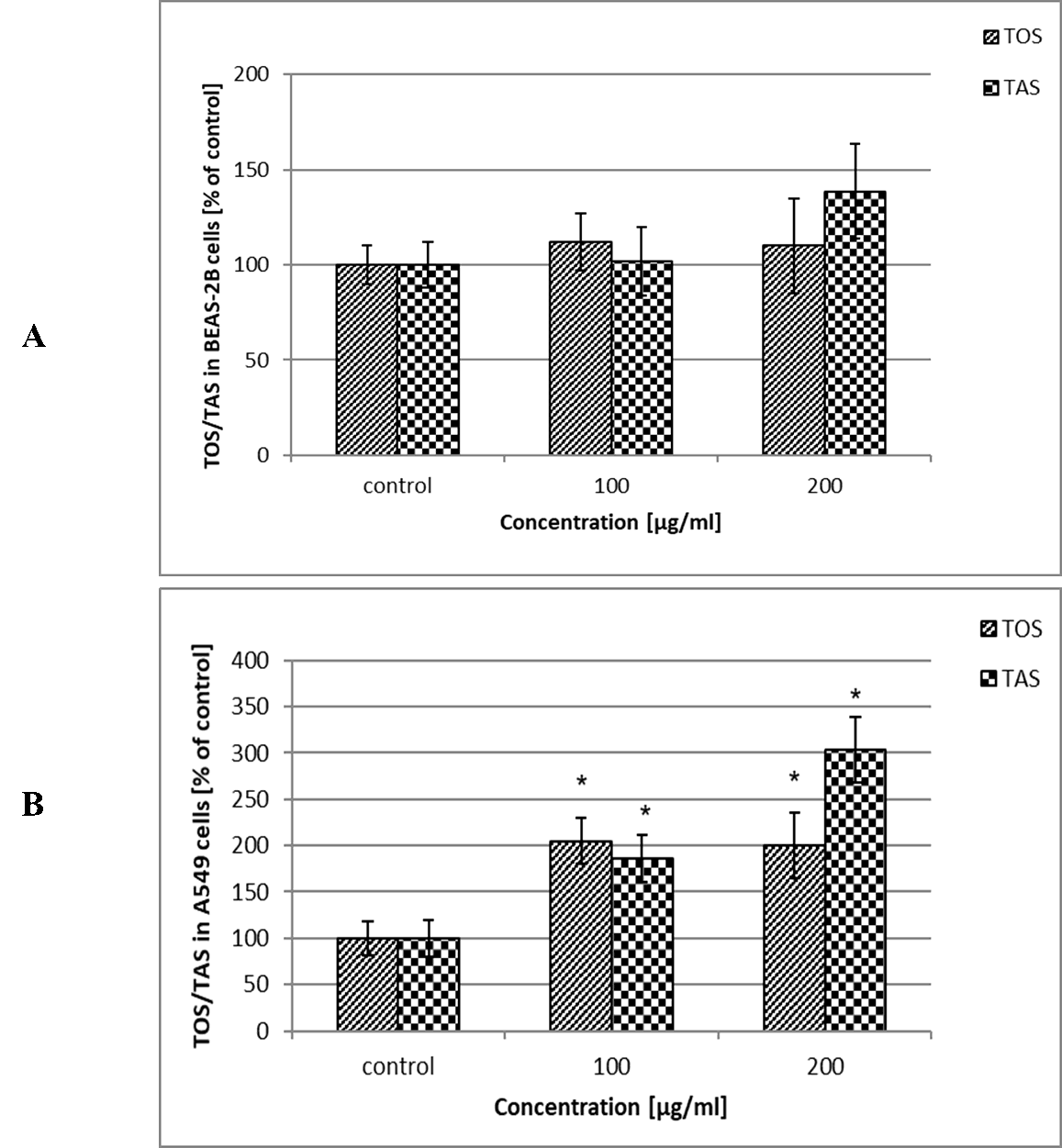

Supplement: Supplementary file 5 — Supplementary material 5 (PNG 63.3 kb) [file 43188_2020_62_MOESM5_ESM.png]

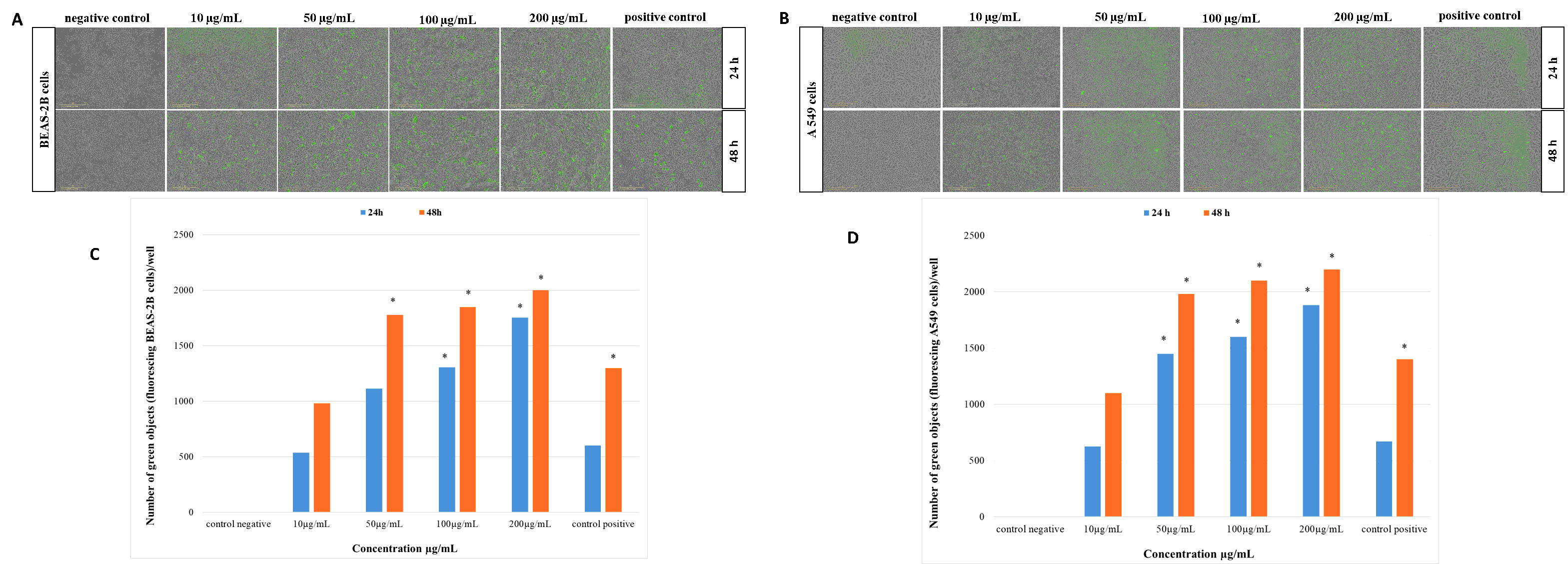

Supplement: Supplementary file 6 — Supplementary material 6 (PNG 315.2 kb) [file 43188_2020_62_MOESM6_ESM.png]

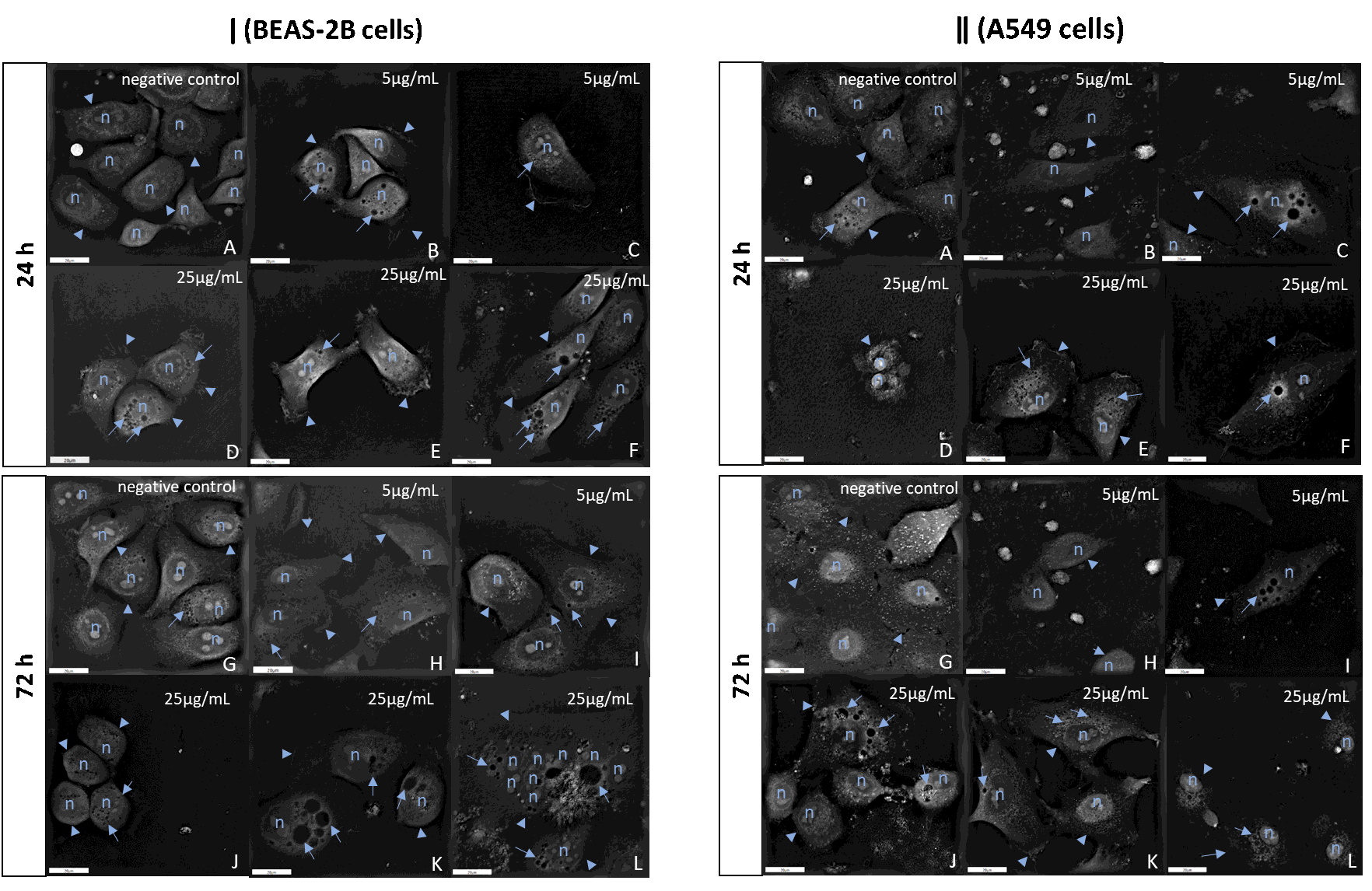

Supplement: Supplementary file 7 — Supplementary material 7 (PNG 272.8 kb) [file 43188_2020_62_MOESM7_ESM.png]
